# Differences in substance abuse knowledge and awareness by year of study among medical students

**DOI:** 10.1186/s12875-026-03386-3

**Published:** 2026-05-25

**Authors:** Elif Deniz-Şafak, Büşra Adlım

**Affiliations:** 1 Department of Family Medicine, Erciyes University School of Medicine, Talas Yolu, Kayseri, 38039 Türkiye; 2 Kızıltepe District Health Directorate, Mardin, Türkiye

**Keywords:** Medical education, Substance use awareness, Knowledge level, Medical students, Public health

## Abstract

**Objective:**

As future healthcare providers, medical students play a critical role in the prevention, early recognition, and management of substance use disorders. However, the extent to which they are adequately prepared for this responsibility remains insufficiently explored. This study aims to evaluate differences in knowledge and awareness according to year of study.

**Methods:**

A total of 607 students (399 first-year and 208 sixth-year) from a medical faculty were included in the study. Data were collected through structured face-to-face interviews using the Substance Abuse Awareness Scale (SAAS) and the Substance Abuse Knowledge Test (SAKT). Descriptive statistics and correlation analyses were performed (*p* < 0.05).

**Results:**

The mean participant age was 20.4 ± 2.6 years, with 54.5% identifying as female. Despite the high rate of formal education (87.0%), variability in knowledge and awareness levels remained evident. The mean scores were 23.0 ± 6.6 for the SAKT and 114.1 ± 12.0 for the SAAS, with the latter demonstrating high internal reliability (Cronbach’s alpha = 0.873). Age positively correlated with SAKT scores and the *Help and Legal Regulations* subscale of the SAAS, whereas no significant correlation was found with other SAAS subscales or the total SAAS score. Female students scored higher on several scales. Grade level significantly influenced awareness across all SAAS subdomains, excluding *Symptoms and Effects of Substance Use*. No significant relationships were found between outcomes and parental education, family structure, or personal history of smoking and alcohol use. Students who had received curricular education on substance use demonstrated significantly higher knowledge levels.

**Conclusion:**

Knowledge and awareness levels differed by year of study, with higher scores observed in senior students. However, targeted improvements—particularly in recognizing symptoms and health effects—are warranted to better equip future physicians for their roles in prevention, early detection, and public health advocacy.

**Supplementary Information:**

The online version contains supplementary material available at 10.1186/s12875-026-03386-3.

## Introduction

Substances are chemical agents that affect brain function and behavior when introduced into the body through various routes. Commonly misused substances include illicit drugs (e.g., marijuana, heroin, cocaine) as well as prescription medications such as amphetamines, benzodiazepines, and other sedative–hypnotics [1]. The World Health Organization (WHO) advocates the use of the term substance dependence rather than drug addiction, as it encompasses a broader and more clinically grounded understanding of the phenomenon [2]. In recent years, substance dependence has emerged as a growing global public health concern, characterized by both an increase in the number of users and a notable decrease in the average age of onset [3].

The increasing prevalence of substance addiction has far-reaching consequences, including adverse effects on family functioning, reduced social cohesion, economic strain, and a rise in crime rates. Accordingly, enhancing public awareness and sensitivity toward substance use disorders has become an urgent public health priority [4]. Healthcare professionals—and particularly medical students, who constitute the future workforce in the prevention, diagnosis, and treatment of such conditions—are expected to possess a higher level of knowledge and awareness regarding substance use than the general population [4, 5].

Although numerous studies have examined healthcare professionals’ awareness of substance use, particularly in adolescent populations, fewer studies have focused on how this knowledge and awareness develop during medical education. In particular, direct comparisons between different stages of medical training are limited. Examining such differences may help to better understand the contribution of medical education to students’ knowledge and awareness in this field.

Therefore, the present study aimed to evaluate and compare the levels of knowledge and awareness regarding substance use and addiction among first- and sixth-year medical students at Erciyes University Faculty of Medicine during the 2023–2024 academic year.

## Materials and methods

### Study design

This descriptive, cross-sectional analytical study was conducted at a single center and included 607 medical students enrolled in the first and sixth years of study at Erciyes University Faculty of Medicine during the 2023–2024 academic year. Data collection was conducted using a researcher-developed questionnaire comprising sociodemographic items and variables related to substance use, along with two validated measurement tools: the Substance Abuse Awareness Scale [5, 6] and the Substance Abuse Knowledge Test [7]. Face-to-face interviews were conducted between December 2023 and March 2024, following the acquisition of informed consent from all participants.

### Inclusion and exclusion criteria

The study included students enrolled in the first or sixth year of medical education at Erciyes University who voluntarily provided informed consent. Exclusion criteria comprised refusal to participate, as well as the presence of any physical or cognitive condition that might compromise data reliability or impede participation in the study.

### Data collection

Data were collected using a questionnaire prepared by the researchers to assess sociodemographic characteristics and substance use–related variables. The questionnaire included items addressing demographic characteristics such as age, gender, marital status, academic year, parental education level, and personal history of alcohol, tobacco, and substance use. In addition, participants were asked open-ended questions about their personal experiences related to substance abuse, including whether they had a close relative with substance dependence, whether they had previously encountered individuals under the influence of substances, and their emotional responses during such encounters. Further items assessed whether participants had received any formal education on substance use during their medical training and whether they were aware of the national drug abuse counseling and support hotline number (Appendix 1).

####  Substance Abuse Awareness Scale (SAAS)

The SAAS, developed in 2018 by Özay Köse and Gül, is designed to assess individuals’ awareness levels regarding substance abuse [5]. It consists of 27 items distributed across four subscales: *Help and Legal Regulations*, *Symptoms and Effects of Substance Use*, *Personal Attitudes and Opinions*, and *Factors Leading to Addiction*.

Each item is rated on a 5-point Likert scale ranging from 1 (*strongly disagree*) to 5 (*strongly agree*), with higher scores indicating greater awareness. Items 18 through 22 are negatively worded and were reverse-coded prior to analysis to ensure scoring consistency. The reliability of the Substance Abuse Awareness Scale (SAAS) has been previously established in the original validation study by Özay Köse and Gül. The Cronbach’s alpha coefficient was reported as 0.882 for the total scale, while the coefficients for the subscales were 0.867, 0.781, 0.710, and 0.647, respectively. These values indicate that the scale demonstrates acceptable reliability. The total possible SAAS score ranges from 27 to 135, with higher scores reflecting a higher level of substance abuse awareness [5, 6].

#### Substance Abuse Knowledge Test (SAKT)

Developed in 2017 by Özay Köse and colleagues, the SAKT assesses secondary school students’ knowledge and awareness of substance use [7]. The test comprises 30 items, each offering three response options: *True*, *False*, and *I don’t know*. Scoring is binary: correct responses receive 1 point, while both incorrect and *I don’t know* responses are scored as 0. The items primarily aim to evaluate the effects of substance dependence on various biological systems and structures. The mean item difficulty of the SAKT was 0.59, and its mean item discrimination index across all items was approximately 0.53. The Kuder–Richardson 20 (KR-20) reliability coefficient of the SAKT was calculated as 0.878. These values suggest that the scale has acceptable reliability. Since there are no established normative data or cut-off value for this instrument, the scores were interpreted based on their respective ranges, with higher scores reflecting greater knowledge and awareness. The total possible SAKT score ranges from 0 to 30, with higher scores reflecting a higher level of substance abuse knowledge and awareness [6–8].

### Ethical approval

Ethical approval for the study was obtained from the Clinical Research Ethics Committee of Erciyes University on November 8, 2023 (Decision No: 2023/726). Furthermore, authorization to administer the questionnaire to medical students was granted by the Dean’s Office of the Erciyes University Faculty of Medicine on October 10, 2023 (Document No: E-49127060-299-523734).

### Statistical analysis

Data were analyzed using SPSS version 28.0 and GraphPad Prism version 10.0.3. Continuous variables were presented as mean±standard deviation and median, while categorical variables were summarized as frequencies and percentages. The assumption of normality was assessed by examining skewness and kurtosis values and by visual inspection of histogram plots.

For the analysis of open-ended responses, qualitative coding was employed. An inductive thematic analysis was conducted following the approach described by Braun and Clarke [9], with codes generated iteratively during the early stages of data analysis and grounded in patterns emerging from participants’ responses. Answers to the question, “If you encountered someone under the influence of an addictive substance before, how did you feel?” were categorized into five thematic groups: “Nothing,” “Sadness,” “Discomfort,” “Fear,” and “Other.”

Similarly, responses to the question “If you previously participated in an event or training related to substance addiction, what was the name of the training?” were categorized into three thematic groups based on content: “Public Health,” “Seminar,” and “Psychiatry.”

In addition, answers to the question “If you took a course or courses on substance addiction during your medical education, what was the name of the course?” were classified into three categories: “Public Health,” “Psychiatry,” and “Pharmacology.”

For normally distributed variables, comparisons between two groups were conducted using Student’s t-test, while one-way ANOVA was employed for comparisons across multiple groups. For non-normally distributed variables, the Mann–Whitney U test and the Kruskal–Wallis test were used for two-group and multiple-group comparisons, respectively. Levene’s test was used to assess variance homogeneity.

To account for multiple comparisons, Bonferroni correction was applied where appropriate, and adjusted *p*-values were reported. A *p*-value of < 0.05 was considered statistically significant after correction.

Correlations between variables were evaluated using Spearman’s rank correlation coefficient.

## Results

The study sample comprised first- and sixth-year medical students from Erciyes University Faculty of Medicine. During the data collection phase, 473 first-year students at Erciyes University Faculty of Medicine were approached before a class session and after an anatomy laboratory session; 74 students declined to participate. Final-year students were contacted at their clinical workplaces, where 312 students were informed about the study; however, 104 declined participations, primarily citing concerns about the personal nature of the questions and demanding work schedules. All participants who agreed to take part received comprehensive information about the study’s purpose and procedures and provided both verbal and written informed consent prior to completing the face-to-face survey. The final sample consisted of 607 participants. Of the participants, 331 (54.5%) were female, 269 (44.3%) were male, and 7 (1.2%) preferred not to disclose their gender.

The mean age of the participants was 20.4 ± 2.6 years. The vast majority were single or separated (*n* = 603, 99.3%), while only four students (0.7%) were married. In terms of academic distribution, 399 students (65.7%) were in their first year, and 208 (34.3%) were in their sixth year.

Regarding lifestyle characteristics, 71 participants (11.7%) reported current cigarette use, whereas 51 participants (8.4%) reported alcohol or illicit substance use.

In terms of exposure to substance use, 299 (49.3%) of respondents reported having at least one relative with a past or current history of substance use. Similarly, 287 (47.1%) indicated that they had previously encountered individuals under the influence of addictive substances.

Regarding educational background, 190 (31.3%) of participants reported prior attendance at an educational activity related to substance addiction, whereas 528 (87.0%) reported receiving formal instruction on the subject during their medical education.

When participants’ attitudes toward individuals with substance dependence were assessed, 351 (57.8%) expressed willingness to diagnose, intervene, and, when necessary, refer patients for further care, whereas only 188 (31.0%) reported willingness to provide long-term treatment.

As part of the statistical analysis, the psychometric properties of the measurement tools were evaluated. The mean score on the Substance Abuse Knowledge Test (SAKT) was 23.0 ± 6.6, with individual scores ranging from 0 to 30. Given the dichotomous nature of SAKT item scoring (0–1), internal consistency was assessed using the Kuder–Richardson 20 (KR-20) coefficient. In the present sample, the KR-20 value was calculated as 0.923, indicating high internal reliability.

The mean score on the Substance Abuse Awareness Scale (SAAS) was 114.1 ± 12.0, with minimum and maximum scores of 54 and 135, respectively. The scale demonstrated high internal consistency, with a Cronbach’s alpha coefficient of 0.873 for the total score.

Correlation analyses were performed to examine the relationship between participants’ age and their scores on the SAKT and SAAS. A statistically significant positive correlation was found between age and the SAKT score (p < 0.001), as well as the Help and Legal Regulations subscale (p < 0.001). No significant associations were observed with the other SAAS subscales (Table 1).

**Table 1:** Correlations between age and scale scores

Variables	Age
*SAKT-Total*	0.173***
*SAAS-Total*	0.079
*Help and legal regulations*	0.145***
*Symptoms and effects of substance use*	-0.024
*Personal attitudes and opinions*	0.044
*Factors contributing to addiction*	0.083

*SAKT *Substance Abuse Knowledge Test, *SAAS *Substance Abuse Awareness Scale

Spearman correlation analysis, p<0.05, ***p<0.001

When scale scores were analyzed according to participants’ characteristics, initial analyses suggested significant gender-based differences across several measures. Female participants scored significantly higher than male participants across all scales. However, after adjustment for multiple comparisons using the Bonferroni method, these differences remained statistically significant only for the SAKT total score, SAAS total score, and the “personal attitudes and opinions” subscale. The previously observed differences in the remaining subscales did not retain statistical significance after correction (Table 2). Participants who selected the option “I do not want to specify” for gender (n = 7) were excluded from the analysis due to the limited sample size, which did not permit meaningful statistical comparison.

**Table 2:** Distribution of scale scores by gender

Variables	Female			Male			Test value	*p*	
	X	sd	M	X	sd	M			Bonferroni *p*
SAKT total*	24.0	5.9	26.0	21.9	7.2	24.0	-4.414	< 0.001	0.006
SAAS-Total*	116.1	10.4	118.0	112.1	12.9	115.0	-3.834	< 0.001	0.006
Help and legal regulations *	38.7	4.2	39.0	37.7	5.2	39.0	-2.173	0.030	0.180
Symptoms and effects of substance use **	34.7	4.0	35.0	34.0	4.7	34.0	1.983	0.048	0.288
Personal attitudes and opinions*	25.4	3.2	26.0	23.7	4.5	25.0	4.507	< 0.001	0.006
Factors contributing to addiction **	17.1	2.6	17.0	16.6	2.7	17.0	2.423	0.016	0.096

*X *Mean, *sd *Standard deviation, *M *Median, *SAKT *Substance Abuse Knowledge Test, *SAAS *Substance Abuse Awareness Scale

* Mann-Whitney U test, ** Student’s t-test

Scale scores differed significantly by academic year. In the initial analysis, no statistically significant difference was observed between first- and sixth-year students in the Symptoms and Effects of Substance Use subscale of the SAAS (Table 3).

**Table 3:** Distribution of scale scores according to year of study

Variables	First-year			Sixth-year			Test value	*p*	
	X	sd	M	X	sd	M			Bonferroni *p*
SAKT total*	22.0	7.2	25.0	24.9	4.7	26.0	-5.365	< 0.001	0.006
SAAS total*	112.7	13.0	115.0	116.8	9.2	118.0	-3.411	< 0.001	0.006
Help and legal regulations*	37.5	5.0	38.0	39.5	4.2	40.0	-4.829	< 0.001	0.006
Symptoms and effects of substance use**	34.2	4.7	35.0	34.5	3.6	35.0	1.983	0.414	1.000
Personal attitudes and opinions*	24.3	4.1	26.0	25.2	3.3	26.0	-2.625	0.009	0.054
Factors contributing to addiction **	16.6	2.9	17.0	17.4	2.2	18.0	2.423	< 0.001	0.006

*X *Mean, *sd *Standard deviation, *M *Median, *SAKT *Substance Abuse Knowledge Test, *SAAS *Substance Abuse Awareness Scale

* Mann-Whitney U test, ** Student’s t-test

After Bonferroni correction, significant differences remained for SAKT total and SAAS total scores, as well as for the Help and Legal Regulations and Factors Contributing to Addiction subscales. However, the difference observed in the Personal Attitudes and Opinions subscale did not remain significant after correction (Table 3).

Regarding the item *“I know what AMATEM (Alcohol and Drug Addiction Treatment Center) is”*, included within the *Help and Legal Regulations* subscale of the SAAS, 298 first-year students (74.6%) and 95 sixth-year students (45.7%) reported that they did not know what AMATEM was.

No statistically significant associations were identified between participants’ scale scores and their parents’ educational attainment. Likewise, scale scores did not significantly vary based on participants’ family structure (nuclear vs. broken), history of tobacco or alcohol use, presence of a relative with substance use issues, or prior exposure to individuals under the influence of addictive substances. Nevertheless, participants who reported previous encounters with individuals under the influence exhibited significantly higher levels of knowledge regarding substance addiction compared to those without such experiences (Z = − 2.366, *p* = 0.018).

When scale scores were compared between participants who had attended an activity or training program related to substance addiction and those who had not, no statistically significant differences were observed in substance addiction awareness, as measured by the SAAS total and subscale scores. In contrast, participants who had engaged in such activities or training exhibited significantly higher knowledge levels, as indicated by their SAKT scores, compared to those who had not participated (Z = − 2.641, *p* = 0.008).

In analyses based on participation in substance dependence–related courses, several differences remained statistically significant after Bonferroni adjustment. These included SAKT total, SAAS total, and the subscales Personal Attitudes and Opinions and Factors Contributing to Addiction. However, no statistically significant differences were found for the Help and Legal Regulations or Symptoms and Effects of Substance Use subscales after correction (Table 4).

**Table 4:** Distribution of scale scores based on participation in a course on substance dependence during medical education

Variables	Taking courses (+)			Taking courses (-)			Test value	*p*	
	X	sd	M	X	sd	M			Bonferroni *p*
SAKT total*	23.3	6.3	25.0	21.1	7.9	24.0	-2.936	0.003	0.018
SAAS-total*	114.7	11.6	116.0	110.3	13.9	113.0	-2.815	0.005	0.030
Help and legal regulations*	38.3	4.8	39.0	37.2	5.2	37.0	-1.837	0.066	0.396
Symptoms and effects of substance use **	34.4	4.2	35.0	33.8	5.0	35.0	1.483	0.224	1.000
Personal attitudes and opinions*	24.8	3.7	26.0	23.1	4.5	25.0	-3.594	< 0.001	0.006
Factors contributing to addiction **	17.0	2.6	17.0	16.1	3.2	16.0	8.672	0.003	0.018

*X *Mean, *sd *Standard deviation, *M *Median, *SAKT *Substance Abuse Knowledge Test, *SAAS *Substance Abuse Awareness Scale

* Mann-Whitney U test, ** Student’s t-test

**Fig. 1:**
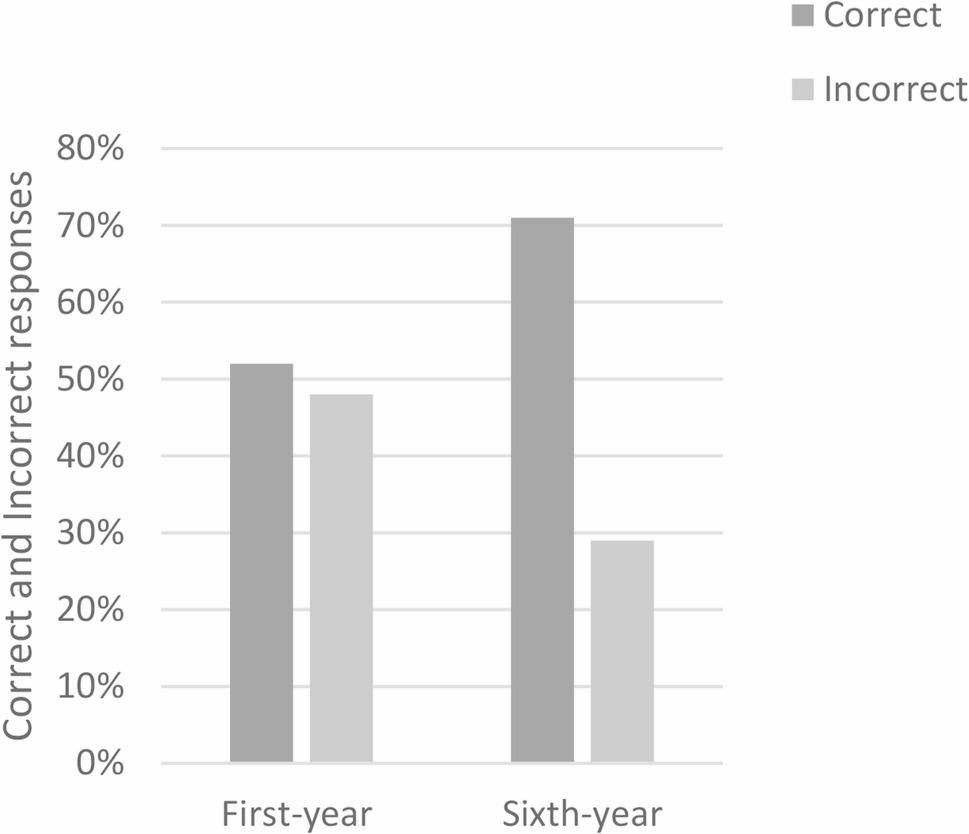
Comparison of correct and incorrect responses to the question “What is the Drug Abuse Counseling and Support Hotline number?” between first-year and sixth-year medical students

When participants were asked, “What is the Drug Abuse Helpline number?”, 57.8% (n = 347) correctly identified the number as “ALO 191.” Comparisons of scale scores between participants who answered the question correctly and those who did not revealed no statistically significant differences. However, the proportion of correct responses was significantly higher among sixth-year students compared to first-year students (χ²=18.3, p < 0.001) (Fig. 1).

**Fig. 2:**
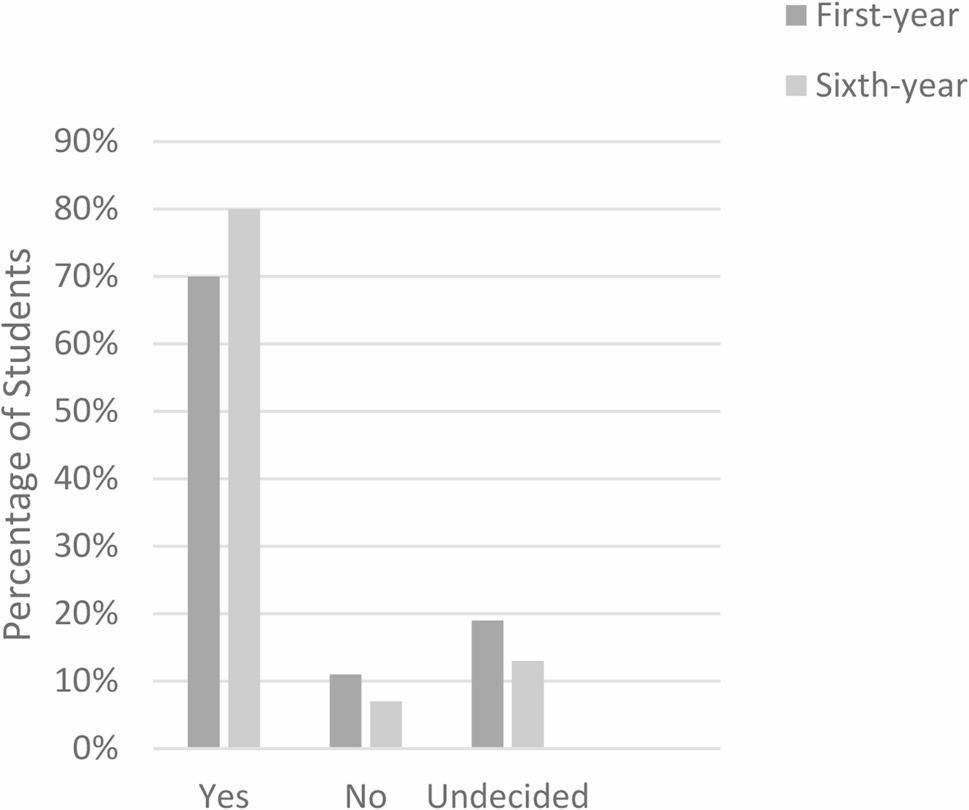
Comparison of responses to the question “Would you like to diagnose, intervene, and, if necessary, refer individuals with substance use disorders?” by academic year (first-year vs. sixth-year medical students)

The proportion of participants who responded “Yes” to the question, “Would you like to diagnose, intervene, and, if necessary, refer individuals with substance use disorders?” was significantly higher among sixth-year medical students compared to first-year students (χ² = 21.8, p < 0.01) (Fig. 2).

A correlation analysis was performed to examine the relationships among participants’ scale scores. The results indicated statistically significant correlations between all scale dimensions. The strongest association was found between the SAAS total score and the Symptoms and Effects of Substance Use subscale (r = 0.777). In contrast, the weakest—though still statistically significant—correlation was observed between the Symptoms and Effects of Substance Use and Personal Attitudes and Opinions subscales (r = 0.184) (Table 5).

**Table 5:** Correlations between scale scores

Variables	SAKT-Total	SAAS-Total	Help and legal regulations	Symptoms and effects of substance use	Personal attitudes and opinions	Factors contributing to addiction
*SAKT-Total*	−	0.350***	0.232***	0.263***	0.250***	0.239***
*SAAS-Total*		−	0.766***	0.777***	0.540***	0.712***
*Help and legal regulations*			−	0.489***	0.283***	0.449***
*Symptoms and effects of substance use*				−	0.184***	0.576***
*Personal attitudes and opinions*					−	0.219***
*Factors contributing to addiction*						−

*SAKT *Substance Abuse Knowledge Test, *SAAS *Substance Abuse Awareness Scale

Spearman correlation analysis, ***p<0.001

**Fig. 3:**
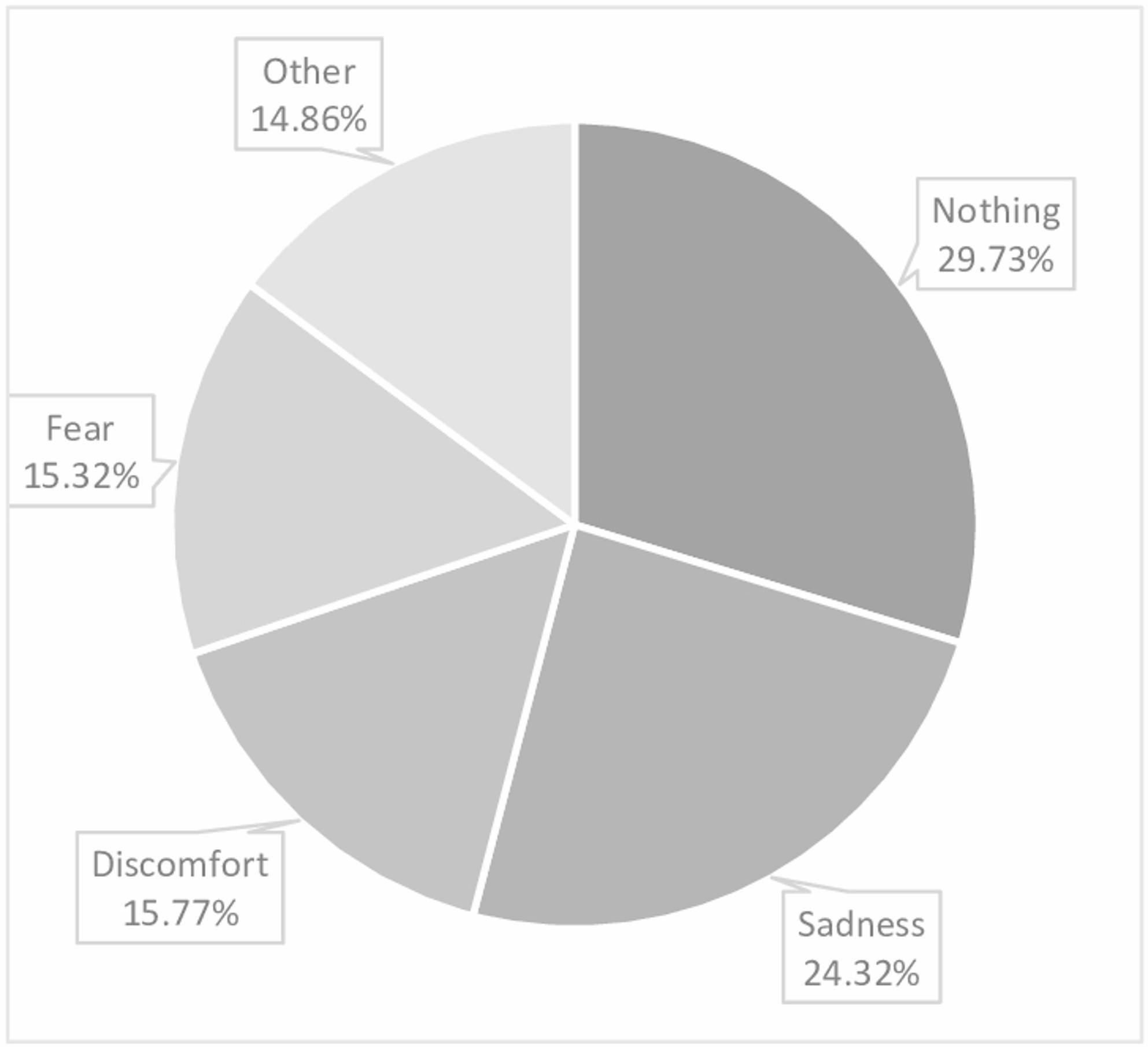
Thematic distribution of responses to the question, “How did you feel when you encountered someone under the influence of drugs?” (*n* = 222)

Participants were asked an open-ended question: “How did you feel when you encountered someone under the influence of substances?” A total of 222 responses were collected and subsequently categorized into predefined thematic codes. The distribution of these thematic responses is presented in Fig. 3.

**Fig. 4:**
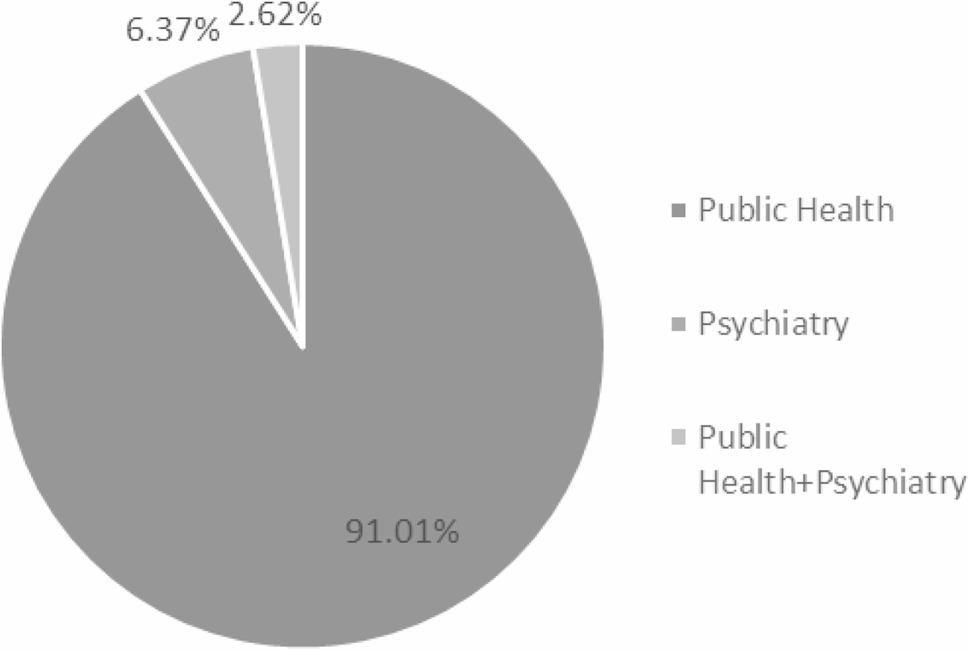
Distribution of responses to the question, “During your medical education, in which internships did you receive training related to substance abuse?” (*n* = 267)

Finally, participants were asked, “During your medical education, in which internships did you receive training related to substance addiction?” A total of 267 participants responded to this question. The majority reported receiving such training during the public health internship (91.01%, n = 243). Similarly, 17 participants (6.37%) reported that they received training during the psychiatry internship, while 7 participants (2.62%) reported receiving training in both public health and psychiatry internships. The distribution of responses is presented in Fig. 4.

## Discussion

This study aimed to assess the level of knowledge and awareness regarding substance use and addiction among first- and sixth-year medical students at Erciyes University Faculty of Medicine during the 2023–2024 academic year.

In the present study, 11.7% of participants reported active smoking, 8.4% reported alcohol consumption, and 0.5% reported drug use. These rates are notably lower than those reported in several previous studies conducted among medical students. For example, Şahin et al. reported smoking and alcohol use rates of 22.2% and 39.6%, respectively [10], while Erşahinoğlu et al. found smoking, alcohol, and drug use rates of 34.6%, 33.3%, and 6.7%, respectively [11]. Similarly, Mayda et al. reported regular cigarette and alcohol use rates of 15.8% and 6.9%, with no reported use of narcotics or stimulants [12].

National data reflect fluctuations in tobacco use in Türkiye, with a decline between 2008 and 2012, followed by a temporary increase in 2014 and a slight decrease thereafter [13]. Alcohol consumption remains relatively low in Türkiye compared to global figures; according to WHO data, 89.1% of the population had never consumed alcohol as of 2016 [14]. In contrast, substance use has shown a rising trend since 2005, with a reported prevalence of substance use disorder reaching 1.54% in 2017 [15]. Variations in prevalence rates across studies may be explained by differences in data collection periods, cultural dynamics, and levels of societal development.

In the present study, 31.3% of participants reported having attended an educational activity related to substance addiction, while 87.0% indicated that they had received formal education on substance use during medical training. Overall, mean SAAS and SAKT scores suggested a generally good level of awareness and knowledge among participants. Although the SAKT has been shown to be valid in adult populations, it was originally developed for a younger group. Its use in a highly educated sample such as medical students may have limited its ability to distinguish higher levels of knowledge and may have contributed to the relatively high scores observed.

When scale scores were analyzed based on whether participants had taken a course on substance dependence, significantly higher scores were observed across all SAAS subscales—except for the *Help and Legal Regulations* and *Symptoms and Effects of Substance Use* subscales—among those who had completed such a course. Additionally, participants who had attended an educational activity related to substance addiction demonstrated significantly higher SAKT scores. However, no significant differences were observed in SAAS total or subscale scores between attendees and non-attendees of such activities. These findings are consistent with those of Öztürk, who reported no significant difference in total SAAS scores based on prior participation in substance use education or activities [16]. In contrast, Bekircan et al. found that 48.6% of university students had not received any education on substance addiction and exhibited low awareness, particularly in the *Personal Attitudes and Opinions* subscale [17].

The differences between the findings of the present study and those reported in the literature may be attributed to the inclusion of students from non-health-related faculties in some studies, as well as limited exposure to substance addiction education among these populations. For instance, Aldemir et al. reported a moderate level of substance addiction awareness among nursing students [4]. However, studies specifically examining substance use awareness among students in health-related fields remain limited. Existing evidence suggests that healthcare professionals may harbor negative attitudes toward individuals with substance use disorders, often viewing substance use as a moral failing or personal weakness rather than a medical condition, and may lack adequate training in this area [18]. Within this context, education—particularly during medical training—appears to play a crucial role in enhancing knowledge and awareness of substance dependence, and may contribute to more informed and compassionate approaches to patients with substance use disorders encountered in clinical settings.

As the participants’ year of study increased, significant differences were observed in SAKT scores and in all SAAS subscales, with the exception of the *Symptoms and Effects of Substance Use* subscale. These findings suggest that medical education contributes positively to substance use awareness—particularly in the domains of *Help and Legal Regulations*, *Personal Attitudes and Opinions*, and *Factors Causing Dependence*—as well as to overall knowledge levels. While prior studies have evaluated the knowledge, attitudes, and perceptions of first-year medical students regarding substance abuse [3], few have examined how these aspects evolve throughout the course of medical education. In this respect, the present findings make a valuable contribution to the literature.

One notable finding of this study is the limited awareness of AMATEM (specialized Alcohol and Drug Addiction Treatment Centers in Türkiye) among medical students. A substantial proportion of first-year students (74.6%) and nearly half of sixth-year students (45.7%) reported that they were unfamiliar with AMATEM. Although awareness was higher among senior students, this gap persisted in the final year. Similar findings have been reported in the literature; in one study, 69.1% of first-year medical students did not know a treatment center for substance use disorders, and among those who did, AMATEM was the most frequently mentioned (57.7%) [3]. Given the central role of these centers in the management and referral of substance use disorders in Türkiye, this gap may be relevant for clinical preparedness and points to an area that could be addressed during undergraduate medical education.

In the present study, substance addiction awareness and knowledge levels differed significantly by gender, with female participants scoring higher than their male counterparts across all scales. Findings from previous studies regarding gender differences remain inconsistent. Some studies reported no significant gender-based differences in awareness or knowledge [16, 19], while others found higher levels of knowledge among female participants [20], or greater awareness among males [21]. The higher awareness and knowledge levels observed among female participants in this study may be associated with greater interest in the topic and a stronger internalization of the consequences of substance use. This interpretation is supported by existing evidence suggesting that women tend to exhibit higher levels of empathy, which may foster increased sensitivity toward substance-related issues [22].

Approximately half of the participants (*n* = 299, 49.3%) reported having a close relative who used addictive substances. No statistically significant differences were observed in scale scores between students with and without a close relative with substance use. Similarly, substance addiction awareness scores did not significantly differ between participants who had encountered someone under the influence of addictive substances and those who had not. Participants who reported encountering individuals under the influence of substances described a range of emotional responses, including indifference, sadness, discomfort, and fear. This variation suggests that such encounters may be perceived differently depending on individual experiences and attitudes. Consistent with these findings, a study conducted at Üsküdar University reported no significant difference in SAAS total scores based on the presence of substance users in an individual’s environment [16].

In contrast, participants who had encountered individuals under the influence of substances demonstrated significantly higher SAKT scores compared to those without such experiences. This finding suggests that, while substance use awareness may be influenced by personal or emotional proximity, knowledge levels appear to be more strongly affected by direct social exposure.

In the present study, more than half of the participants reported that they would be willing to diagnose, intervene, and refer individuals with substance use disorders. This willingness was higher among final-year students, which may reflect the impact of increased exposure to substance-related content during medical training.

In contrast, willingness to provide long-term care appeared to be more limited. In Türkiye, a considerable proportion of individuals seeking psychiatric services first present to primary care, yet only a smaller proportion receive treatment [23]. This may indicate that physicians without specialized training feel less prepared to manage substance use disorders over the long term. Strengthening both undergraduate and postgraduate training in this area may help improve continuity of care in primary care settings [24].

The relatively low willingness to provide long-term care observed in this study may also be influenced by participants’ career preferences, particularly among those not intending to specialize in psychiatry or family medicine—fields that typically adopt a more holistic and longitudinal approach to patient care. Nevertheless, some encouraging attitudes were also observed. Many participants reported that they would be willing to communicate with individuals with substance use disorders for preventive purposes and to do so in a nonjudgmental manner. Most participants also indicated that they would refer such individuals for medical evaluation if encountered as patients, which is consistent with recommended clinical practices [24, 25].

Participants who reported having received education on substance addiction were further asked to indicate during which internships this training took place. The responses suggest that such training is delivered predominantly during the public health internship, with more limited exposure during psychiatry training or across multiple rotations.

At Erciyes University Faculty of Medicine, first-year students are required to complete the public health internship, while the psychiatry internship is undertaken in the fifth year. Given the limited time available for participants to complete the questionnaire, recall bias may have influenced their responses regarding the internship during which they received substance addiction training. Thus, the predominance of responses identifying the public health internship may reflect better recall of content delivered during that stage, rather than greater exposure to substance addiction education within that particular internship.

The findings of this study have several implications for both medical education and primary care practice. The increase in knowledge and awareness observed with advancing years of medical training suggests that medical education contributes positively in this area. However, the relatively low willingness to provide long-term care suggests that knowledge alone may not be sufficient to ensure confidence in the management of substance use disorders.

These findings suggest that incorporating more practice-oriented and continuous training on substance use disorders into medical curricula may be beneficial. In addition, considering that primary care physicians are often the first point of contact for individuals with substance use disorders, improving both knowledge and attitudes during medical education may facilitate earlier recognition, appropriate referral, and more effective management in primary care settings.

In the initial analyses, several statistically significant differences were observed across scale scores. However, after adjustment for multiple comparisons using the Bonferroni correction, some of these differences—particularly at the subscale level—were no longer statistically significant. This indicates that these findings should be interpreted with caution.

Despite this, the overall results of the study remained largely unchanged. The differences observed in total knowledge and awareness scores (SAKT and SAAS) according to gender, academic year, and participation in substance use–related education persisted after adjustment. This suggests that the main findings of the study are consistent and not solely dependent on multiple testing.

It should also be noted that the Bonferroni correction is a conservative method, especially when applied to related subscales derived from the same measurement tool. While this approach helps to control Type I error, it may increase the likelihood of Type II error by reducing sensitivity to detect real differences. Therefore, subscale-level findings that did not remain statistically significant after correction should not be disregarded entirely and may still be relevant, particularly in the context of clinical interpretation and future research.

### Strengths of the study

- This study addresses an important gap in the literature, as research examining substance addiction awareness among medical students remains limited. To the best of our knowledge, this is the first study conducted in Kayseri to explore this topic specifically among medical students.
- The study achieved a high response rate, with participation from 84.3% of first-year students and 66.6% of sixth-year students. This enhances the representativeness of the sample and supports the reliability of the findings within the target population.

### Limitations of the study

- The study did not assess participants’ personal interest or voluntary engagement with substance addiction–related topics outside the formal medical curriculum, which may have influenced their knowledge and awareness levels.
- The questionnaires were administered before or after scheduled classes and during study hours, which may have affected participants’ focus and the accuracy of their responses.
- As the survey included personal and potentially sensitive questions, the risk of response bias or underreporting cannot be ruled out. The lower participation rate among final-year students—partly related to the personal nature of the questions and demanding schedules—may have introduced selection bias, as those who declined may differ systematically from those who participated.
- Another limitation of this study is that, although the SAKT has been validated in adult populations, it was originally developed for secondary school students. Therefore, its use in a highly educated group such as medical students may have limited its ability to discriminate between higher levels of knowledge, potentially leading to a ceiling effect, which may be reflected in the relatively high scores observed.
- Since the data were collected solely from first- and sixth-year medical students at Erciyes University Faculty of Medicine during the 2023–2024 academic year, the generalizability of the findings to other institutions, year levels, or geographic regions is limited.

## Conclusion

This study indicates that medical students generally have adequate knowledge and awareness of substance addiction, with higher levels observed among female students. Parental education and personal substance use were not associated with these outcomes.

Although most participants reported receiving formal education on the topic, willingness to provide long-term care remained limited. Differences by year of study suggest that knowledge and awareness tend to increase over the course of medical education; however, given the cross-sectional nature of the study, these findings reflect associations rather than causation.

## Supplementary Information

Supplementary Material 1.

## Data Availability

Study data and materials can be requested from the corresponding author if needed.
