# Supplementary material for: Differences in substance abuse knowledge and awareness by year of study among medical students
Source: BMC Prim Care. 2026 May 25;27:280. doi: 10.1186/s12875-026-03386-3 (PMC13411981; doi:10.1186/s12875-026-03386-3)
Supplement: Supplementary file 1 — Supplementary Material 1. [file 12875_2026_3386_MOESM1_ESM.pdf]

## Sociodemographic and Selected Characteristics Form

**1. Age: …….**

**2. Gender:** ( ) Female ( ) Male ( ) Prefer not to say

**3. Marital Status:** ( ) Single ( ) Married ( ) Divorced/Widowed

**4. Year of Study:** ( ) 1st year ( ) 6th year

**5. Mother's Education Level:** ( ) Primary School ( ) Middle School ( ) High School
( ) University ( ) Master's Degree

**6. Father's Education Level:** ( ) Primary School ( ) Middle School ( ) High School
( ) University ( ) Master's Degree

**7. Family Status:** ( ) Intact family – living with parents
( ) Intact family – living away from parents
( ) Separated family – living with one parent
( ) Separated family – not living with either parent

**8. Smoking Status:** ( ) Never smoked
( ) Smoked at least once
( ) Currently smoking
( ) Used before, but quit

**9. Alcohol Use:** ( ) Never used
( ) Used at least once
( ) Currently using
( ) Used before, but quit

**10. Substance Use:** ( ) Never used
( ) Used at least once
( ) Currently using
( ) Used before, but quit

**11. Do you have a relative or someone close who has used or is using addictive substances?**

( ) Yes    ( ) No

**12. Have you ever encountered someone under the influence of substances?** ( ) Yes    ( ) No

**If yes, what did you feel? ……………………………………**

**13. Have you ever participated in an event or training about substance addiction?** ( ) Yes    ( ) No

**If yes, what was the name of the training? ……………………………………**

**14. During your medical education, have you taken any course(s) related to substance addiction?**
( ) Yes    ( ) No

**If yes, what was the name of the course? ……………………………………**

**15. What is the phone number of the Drug Addiction Counseling and Support Line?**

( ) 156    ( ) 177    ( ) 181    ( ) 191    ( ) 114

**16. Would you like to diagnose and intervene in patients with substance addiction?**

( ) Yes    ( ) No    ( ) Undecided

**17. Would you like to work with a substance-addicted patient in the long term?**

( ) Yes    ( ) No    ( ) Undecided

**18. Would you conduct interviews with patients using substances to prevent addiction?**

( ) Yes    ( ) No    ( ) Undecided

**19. Could you speak with a substance user without judging or blaming them?**

( ) Yes    ( ) No    ( ) Undecided

**20. Since the general health condition of substance users may be poor, would you refer them for health screening?**

( ) Yes    ( ) No    ( ) Undecided
